# Potential Impact of Cancer Susceptibility Genes on Lung Cancer Metastasis

**DOI:** 10.1155/2022/1516946

**Published:** 2022-04-18

**Authors:** Jiaqing Wang, Bin Peng, Xuefeng Sun, Peikun Ding, Shixuan Li, Guofeng Li, Xiaoshun Shi, Guangsuo Wang

**Affiliations:** ^1^Department of Thoracic Surgery, The First Affiliated Hospital of Southern University of Science and Technology, Shenzhen People's Hospital, Shenzhen 518020, China; ^2^Department of Thoracic Surgery, Nanfang Hospital, Southern Medical University, Guangzhou 510000, China

## Abstract

**Background:**

Studies of prognosis-related molecular markers are an important tool to uncover the mechanism of tumour metastasis. Cancer susceptibility gene testing is an important tool for genetic counselling of cancer risk. However, the impact of lung cancer susceptibility genes (LCSGs) on lung cancer metastasis and prognosis has not been well studied.

**Methods:**

The list of lung cancer susceptibility genes was retrospectively analysed and updated. After expression profiling and functional analysis, LCSG-based signatures for prognosis were identified by Cox regression and LASSO regression analyses. For translational purposes, nomograms integrating LCSGs and clinical characteristics were constructed.

**Results:**

A total of 301 LCSGs were employed for modelling. For lung adenocarcinoma (LUAD) and lung squamous cell carcinoma (LUSC), 10-gene and 7-gene signatures were created and independently validated. The LCSG-based risk score could stratify LUAD survival (univariate: hazard ratio (HR) = 1.076, 95% confidence interval (CI) = 1.049–1.103, *P* < 0.001; multivariate: HR = 1.066, 95% CI = 1.037–1.095, *P* < 0.001) and LUSC survival (univariate: HR = 1.149, 95% CI = 1.066−1.239, *P* < 0.001; multivariate: HR = 1.129, 95% CI = 1.038−1.228, *P* = 0.005). One of the processes affected by differentially expressed genes in both LUAD and LUSC was the negative regulation of epithelial cell differentiation.

**Conclusions:**

Overall, novel LCSG-based gene signatures for LUAD and LUSC were constructed. These findings could expand the understanding of the impact of LCSG expression on cancer metastasis and prognosis.

## 1. Background

Lung cancer is a type of malignant disease of the respiratory system. Studies of lung cancer susceptibility genes (LCSGs) are focusing on understanding the aetiology, screening, prevention, and treatment of lung cancer-susceptible populations. With the development and application of next-generation sequencing technology, increasing numbers of LCSGs have been identified [1, 2]. Additionally, previous studies have shown that some LCSGs are associated with lung cancer prognosis [3–5]. However, current studies have not summarized the list of LCSGs, leaving the systematic assessment of their overall functions and impact on lung cancer prognosis as an under-researched area.

The mechanism of an LCSG that causes lung cancer varies from gene to gene. For example, X-ray repair cross-complementing (*XRCC*) is associated with lung cancer risk [6, 7] by affecting the ability to repair damage caused by carcinogens. In addition, *CYP450* family genes, which play critical roles in processing chemical carcinogens in vivo, are associated with lung cancer susceptibility [8, 9]. However, either abnormal metabolism or impaired DNA function caused by a single gene may not reflect a general mechanism of lung cancer susceptibility, masking critical targets for prevention.

Cancer metastasis is an important factor affecting prognosis. Some LCSGs are associated with prognosis, but the evidence is mostly at the single-gene level. For example, *XRCC1* is reported to be linked to the susceptibility and prognosis of lung squamous carcinoma [4]. In addition, LCSG *TERT* has been linked to the prognosis of early-stage non-small cell lung cancer (NSCLC) [10]. Currently, the prognostic role of LCSGs and the impact of metastasis have not been systematically reported, so their clinical application is mostly limited in the prediction of cancer risk.

Given the current findings, we first collected a comprehensive set of LCSGs to provide an updated list for clinical genetic counselling. Next, we employed Gene Ontology (GO) and Kyoto Encyclopedia of Genes and Genomes (KEGG) analyses, as well as Gene Set Enrichment Analysis (GSEA), to thoroughly analyse the common functions of the LCSGs with the goal of identifying general preventive targets. Finally, in addition to single-gene analysis, Cox proportional hazards regression analysis and the least absolute shrinkage and selection operator (LASSO) were used to mine LCSGs related to lung cancer prognosis. Then, a clinically applicable nomogram model was constructed, maximizing the translational yield of LCSGs.

## 2. Methods

### 2.1. Identification of LCSGs

LCSGs were identified from 3 independent resources: mapped single-nucleotide polymorphisms (SNPs) associated with lung cancer in the genome-wide association studies (GWAS) catalogue (https://www.ebi.ac.uk/gwas/), previously annotated LCSGs [11], and literature review (http://www.ncbi.nlm.nih.gov/pubmed/). For the literature review, candidate genes associated with lung cancer were queried with the terms lung cancer (MeSH) and susceptibility (MeSH). Initially, the titles and abstracts of these publications were reviewed and genetic association studies of lung cancer were retained. To obtain reliable genes with SNPs associated with lung cancer risk, only those with a significance level of *P* < 10^−8^ together with independent literature support were included in the current study.

### 2.2. Expression Profiles of LCSGs

Based on the identified LCSGs, we retrieved gene expression data from The Cancer Genome Atlas (TCGA) Genomic Data Commons (GDC) (2019-12-06) and the Broad Institute Cancer Cell Line Encyclopedia (CCLE) database (RNA sequencing gene expression data for 1019 cell lines in fragments per kilobase of exon model per million mapped reads) [12]. We displayed the LCSG expression profiles by the R package pheatmap and the overlapping genes by the online Venn diagrams tool (http://bioinformatics.psb.ugent.be/webtools/Venn/).

### 2.3. Functional Enrichment Analysis of the LCSGs

We used clusterProfiler to analyse the functional enrichment of the LCSG list [13]. The associated functional categories were assessed using GO and KEGG. Significant pathways were defined as GO and KEGG enrichment pathways with *P* values and *q* values less than 0.05. GSEA was also used to compare the signalling pathways of the high-risk and low-risk groups.

### 2.4. Protein–Protein Interactions of LCSGs

The permutation type of the phenotype was chosen, and the number of permutations was set to 1000. The Search Tool for the Retrieval of Interacting Genes/Proteins (STRING) (https://string-db.org/) was used to predict the protein–protein interaction network. In brief, the LCSGs were used as an input list; then, the multiple protein method was applied under default settings. Finally, Cytoscape software was used for network visualization.

### 2.5. Survival Analysis of the LCSGs

Corresponding clinical information was also retrieved from the TCGA GDC (2019-12-06). We applied Kaplan–Meier analysis to each LCSG and then performed a meta-analysis by the R package meta. Heterogeneity among genes were evaluated with Cochran's *Q* test and the *I*^2^ statistic. For a dataset with *I*^2^ ≥ 50% (lung adenocarcinoma (LUAD) susceptibility genes significantly associated with overall survival (OS)), the random effects model was applied, while for a dataset with *I*^2^ < 50% (lung squamous cell carcinoma (LUSC) susceptibility genes significantly associated with OS), the fixed effects model was chosen for the calculation of the combined effect. The overlapping survival-associated LCSGs of both cancer types were visualized via a Venn diagram online tool at http://bioinformatics.psb.ugent.be/webtools/Venn/.

### 2.6. Prognostic Model

We first used univariate Cox regression to analyse which LCSGs were related to patient survival for the preparation of the model. The patients in the TCGA-LUAD and TCGA-LUSC cohorts were then randomly divided into training and test sets in a 6 : 4 ratio. Then, using LASSO regression, genes correlated with prognosis (*P* < 0.05) from the univariate Cox regression model in the training set were chosen to build a prognostic model. The gene expression of each gene was used to create a risk score formula, which was then weighted, and patients were separated into two groups: high risk and low risk. Kaplan–Meier analysis was used to analyse the differences in survival between the two groups, and the log-rank test was used to compare them. The accuracy of the model prediction was investigated using a receiver operating characteristic (ROC) curve.

### 2.7. Statistical Analysis

R was used to conduct all statistical analyses (version 3.6). All statistical tests were two sided, and statistical significance was defined as *P* < 0.05.

## 3. Results

### 3.1. Updated List of LCSGs

**Figure 1:**
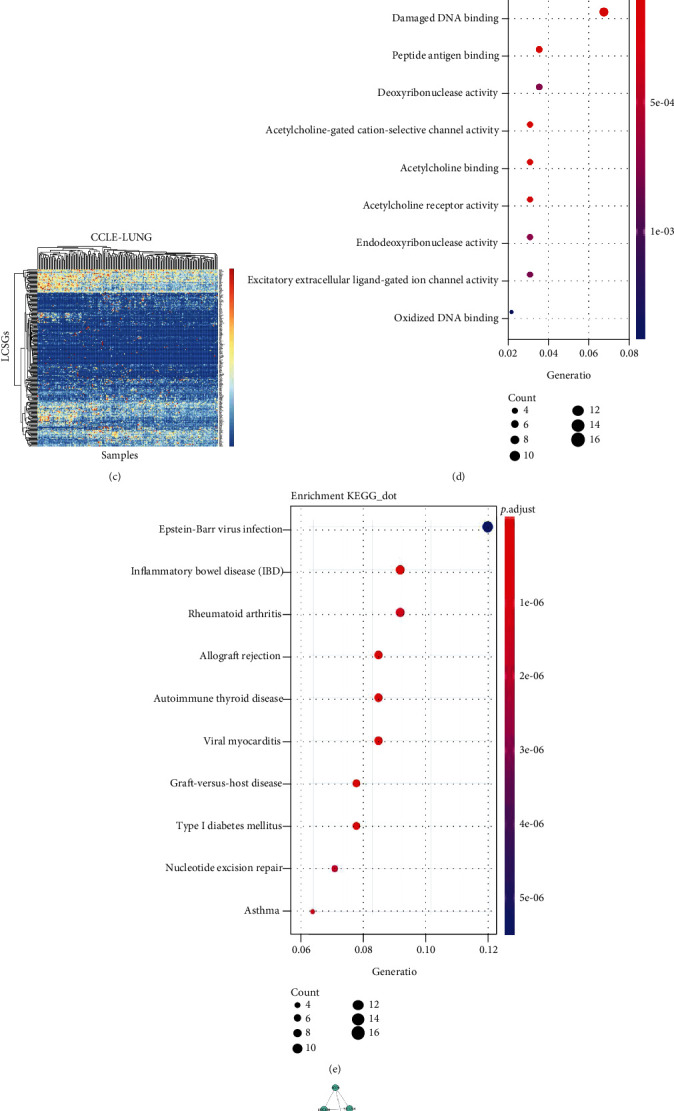
Expression profiles and functions of the LCSGs. The expression profile of current LCSGs in the (a) TCGA-LUAD cohort, (b) TCGA-LUSC cohort, and (c) CCLE lung cancer cell line cohort. Functional analysis of the LCSGs by (d) GO, (e) KEGG, and (f) protein–protein interaction analyses.

Based on the current findings from the GWAS catalogue and literature review, a total of 301 genes were reported as LCSGs after unification. The genes, predisposed lung cancer subtypes, and sources of evidence are reported in Table S1. We observed a subset of genes with low expression across lung cancer cell lines and tissues (Figure 1(a), LUAD cohort; Figure 1(b), LUSC cohort; and Figure 1(c), CCLE lung cancer cell line cohort). Next, an LCSG-specific network revealed that a majority of the genes have close internal crosstalk. Functional enrichment analysis of these genes showed that the GO terms were enriched in DNA binding, peptide antigen binding, acetylcholine-gated cation-selective channel activity, and excitatory extracellular ligand-gated ion channel activity (Figure 1(d)). KEGG analysis showed that these genes were associated with multiple immune diseases, such as rheumatoid arthritis, autoimmune thyroid disease, inflammatory bowel disease, and asthma (Figure 1(e)). The diverse functions of these genes reveal the complexity of genetic factors predisposing individuals to lung cancer. Our protein–protein interaction analysis indicated that a complex network is affected by lung cancer–susceptible genetic factors (Figure 1(f)).

We used Kaplan–Meier analysis based on the median expression level of the retrievable LCSGs to more deeply study the link between LCSGs and lung cancer survival. After analysing the impact of LCSG expression on lung cancer survival, a meta-analysis was performed to investigate the general effect. As expected, not all LCSGs were associated with prognosis, with 31 out of 195 (15.9%) genes in LUAD and 19 out of 196 (9.7%) genes in LUSC, and overall, these genes did not have an impact on prognosis (Figure S1a: LUAD and S1B: LUSC). Furthermore, in both LUAD and LUSC, a minor overlap of LCSGs was linked to survival (Figure S1c). Since the impact of LCSGs on survival is different in terms of pathohistological categories, we developed separate prognostic prediction models for NSCLC patients.

### 3.2. Functions of the Differentially Expressed LCSGs

**Figure 2:**
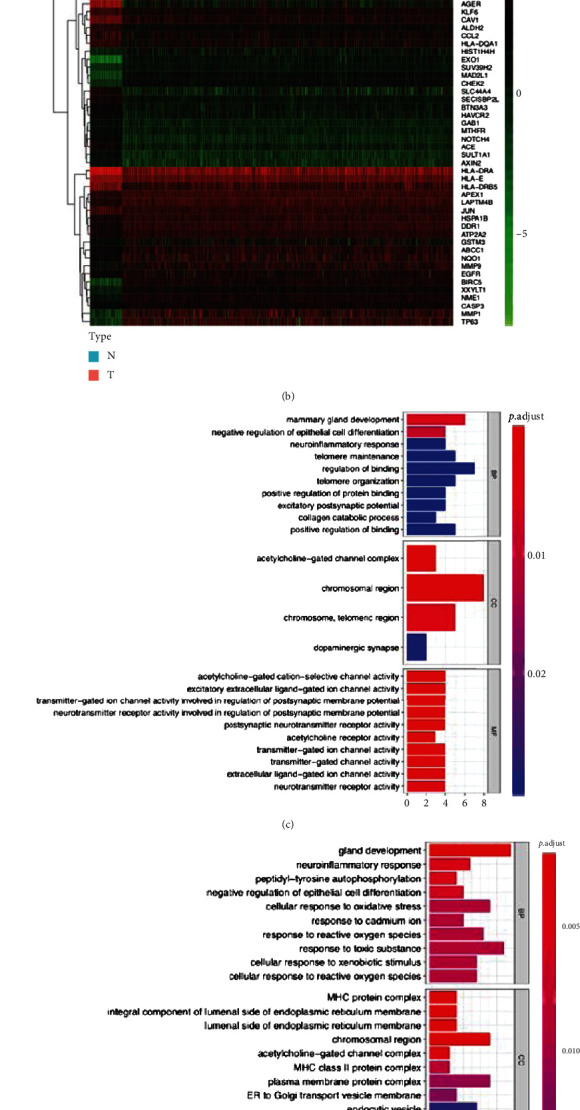
Histology-specific functional analysis of the LCSGs. Differentially expressed genes in the (a) TCGA-LUAD cohort and (b) TCGA-LUSC cohort. GO analysis of the LCSGs in the (c) TCGA-LUAD cohort and (d) TCGA-LUSC cohort. KEGG analysis of the LCSGs in the (e) TCGA-LUAD cohort and (f) TCGA-LUSC cohort.

All LCSGs were first subjected to differential expression analysis, which showed that 28.2% and 36.1% of the LCSGs were differentially expressed in LUAD and LUSC, respectively (Figure 2(a), LUAD, and Figure 2(b), LUSC). Then, the identification of genes significantly associated with the OS of TCGA-LUAD and TCGA-LUSC was performed by univariate Cox regression analysis, which resulted in 21 and 13 genes, respectively (Table S2). Functional enrichment analysis was applied to study gene function. We noticed that regulation of epithelial cell differentiation, excitatory extracellular ligand-gated ion channel activity, acetylcholine-gated cation-selective channel activity, and acetylcholine receptor activity in the GO term molecular function (Figure 2(c)) and rheumatoid arthritis in KEGG (Figure 2(e)) were shared in the abovementioned analysis, suggesting that these pathways play an essential role in LCSG-induced LUAD prognosis. Similarly, the CSGs in LUSC were enriched in acetylcholine-gated cation-selective channel activity, acetylcholine receptor activity, and excitatory extracellular ligand-gated ion channel activity (overlapping with LUAD as well) in GO term molecular function (Figure 2(d)) and asthma, autoimmune thyroid disease, allograft rejection, type I diabetes mellitus, rheumatoid arthritis, and IBD in KEGG (Figure 2(f)). Notably, negative regulation of epithelial cell differentiation is one of the common pathways affected by differentially expressed genes in both LUAD and LUSC.

### 3.3. Development of LCSG-Based Prognostic Signatures

**Figure 3:**
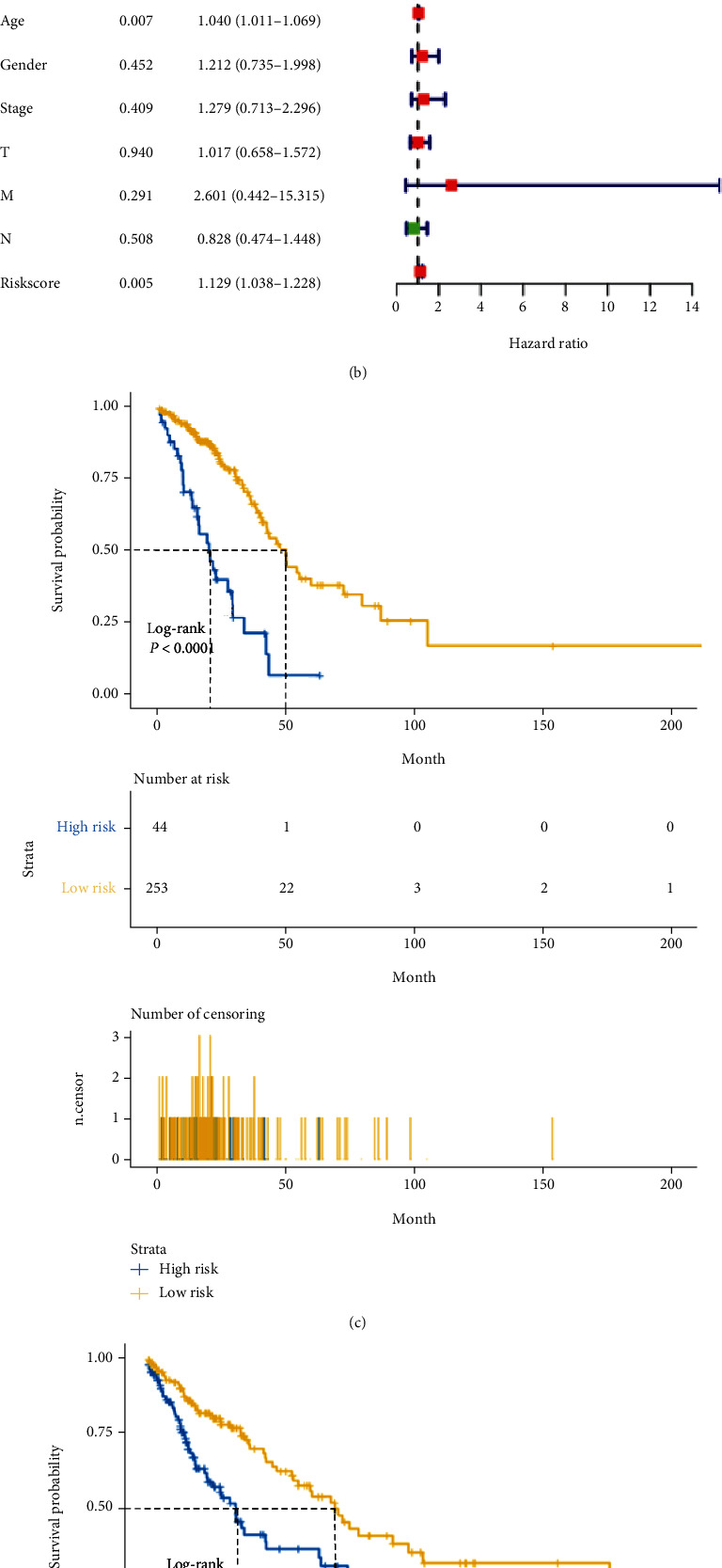
The associations of the LCSG-specific signature with clinical characteristics. Univariate Cox regression and multivariate Cox regression analyses of the (a) TCGA-LUAD and (b) TCGA-LUSC cohorts. The high-risk scores in both the (c) TCGA-LUAD cohort and (d) TCGA-LUSC cohort were an indicator of poor overall survival. The ROC curves for the (e) TCGA-LUAD cohort and (f) TCGA-LUSC cohort were used to examine the sensitivity and specificity of the 1-year, 3-year, and 5-year survival predictions.

Next, the regression coefficients from LASSO Cox regression analysis were applied to establish an LCSG prognostic signature. We narrowed the prognostic genes down to 10 and 7 genes for TCGA-LUAD (Figure S2a and S2b) and TCGA-LUSC (Figure S2c and S2d), respectively. Figure S2e depicts the distribution of the patients' risk scores for LUAD and S2F for LUSC. As shown by univariate and multivariate analyses, the prognostic risk score was associated with LUAD survival (univariate: hazard ratio (HR) = 1.076, 95% confidence interval (CI) = 1.049−1.103, *P* < 0.001; multivariate: HR = 1.066, 95% CI = 1.037−1.095, *P* < 0.001; Figure 3(a)) and LUSC survival (univariate: HR = 1.149, 95% CI = 1.066−1.239, *P* < 0.001; multivariate: HR = 1.129, 95% CI = 1.038−1.228, *P* = 0.005; Figure 3(b)). In both the TCGA-LUAD and TCGA-LUSC cohorts, patients with low risk scores survived longer than those with high risk scores in the Kaplan–Meier survival analysis (Figure 3(c): LUAD and Figure 3(d): LUSC). According to the ROC curves, the LCSG-specific risk score was effective in predicting 1-, 3-, and 5-year prognosis for lung cancer patients and the highest area under the curve (AUC) values of the risk score were 0.718 for 1-year LUAD prognosis and 0.679 for 3-year LUSC prognosis (Figure 3(e): LUAD and Figure 3(f): LUSC).

### 3.4. Genetic Functions of the LCSGs in the Prognostic Model

**Figure 4:**
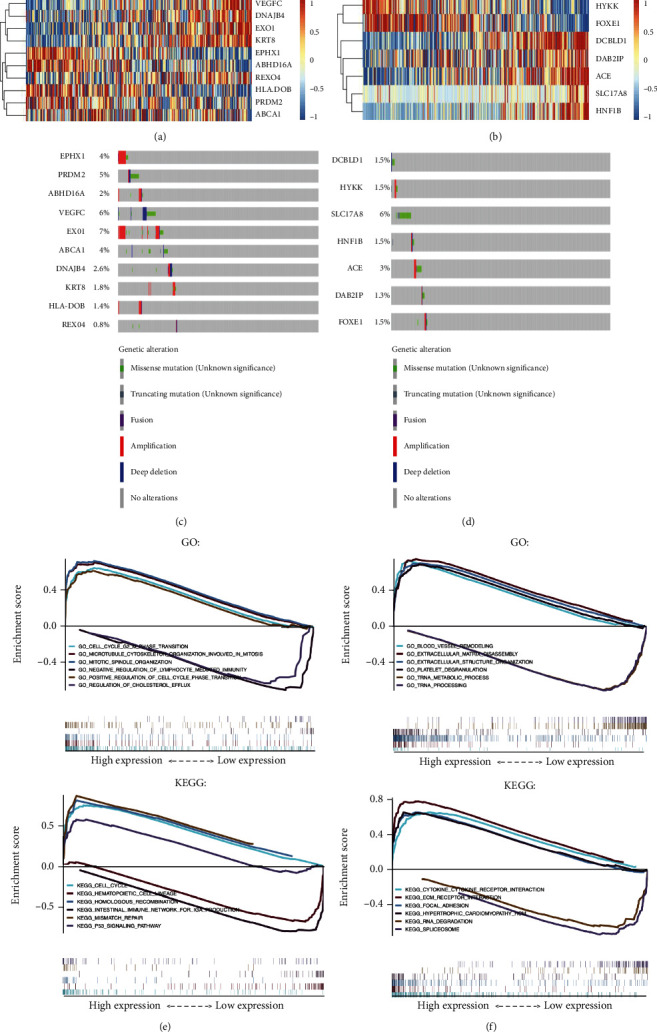
Genetic characteristics and functional analysis of the LCSG-specific signature. Gene expression profiles of the LCSG-specific signature for (a) TCGA-LUAD and (b) TCGA-LUSC. Genetic alteration profiles of the LCSG-specific signature for (c) TCGA-LUAD and (d) TCGA-LUSC. Gene set enrichment analysis for (e) TCGA-LUAD and (f) TCGA-LUSC.

The full name, genomic location, associated disease other than lung cancer [14], and risk coefficients of the genes in the model are shown in Table 1. After profiling the expression heat map of the prognostic LCSGs in the LUAD cohort (Figure 4(a)) and LUSC cohort (Figure 4(b)), the genetic alteration rate of the prognostic LCSGs was also studied, showing rates from 0.8% to 7% in LUAD and (Figure 4(c)) 1.3% to 6% in LUSC (Figure 4(d)). GSEA showed that altered signature genes were mainly associated with cell cycle function in LUAD (Figure 4(e)), while the enriched signalling pathways were more heterogeneous in LUSC (Figure 4(f)). These findings reveal the different roles of LCSGs in lung cancer survival and further support that the genetic liability contributed by the LCSGs of the different pathohistological lung cancer subtypes should also be considered.

### 3.5. Validation of the LCSG-Specific Prognostic Signatures

**Figure 5:**
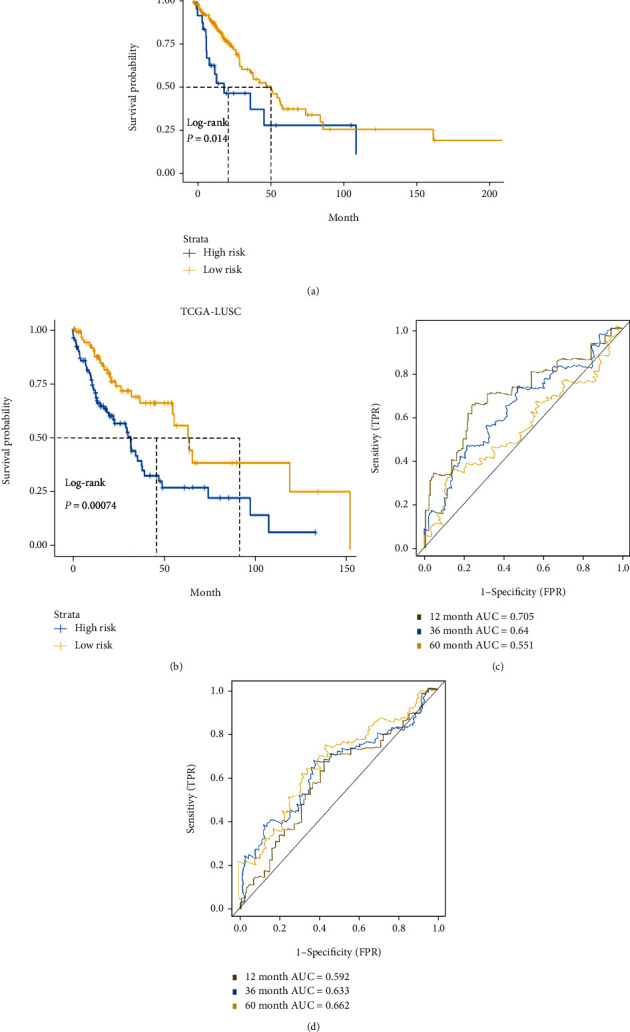
Validation of the LCSG-specific signature. Kaplan–Meier survival curves of overall survival in the high- and low-risk groups defined by the LCSG-specific model for the (a) TCGA-LUAD cohort and (b) TCGA-LUSC cohort were plotted. The areas under the ROC curve of the LCSG-specific model for predicting 1-year, 3-year, and 5-year OS were calculated.

We further evaluated the predictive power of our model in the TCGA-LUAD and TCGA-LUSC validation sets. By using the constructed equation, the risk score of each patient in the validation set was calculated (Figure S3a: LUAD and S3b: LUSC), and then, the patients were grouped based on their risk score to verify its association with survival status. Both the TCGA-LUAD (Figure 5(a)) and TCGA-LUSC (Figure 5(b)) cohorts revealed that patients with low-risk scores had better survival than those with high-risk scores. ROC analyses were used to evaluate the model (Figure 5(c): LUAD and Figure 5(d): LUSC).

### 3.6. LCSG-Specific Nomogram Model

**Figure 6:**
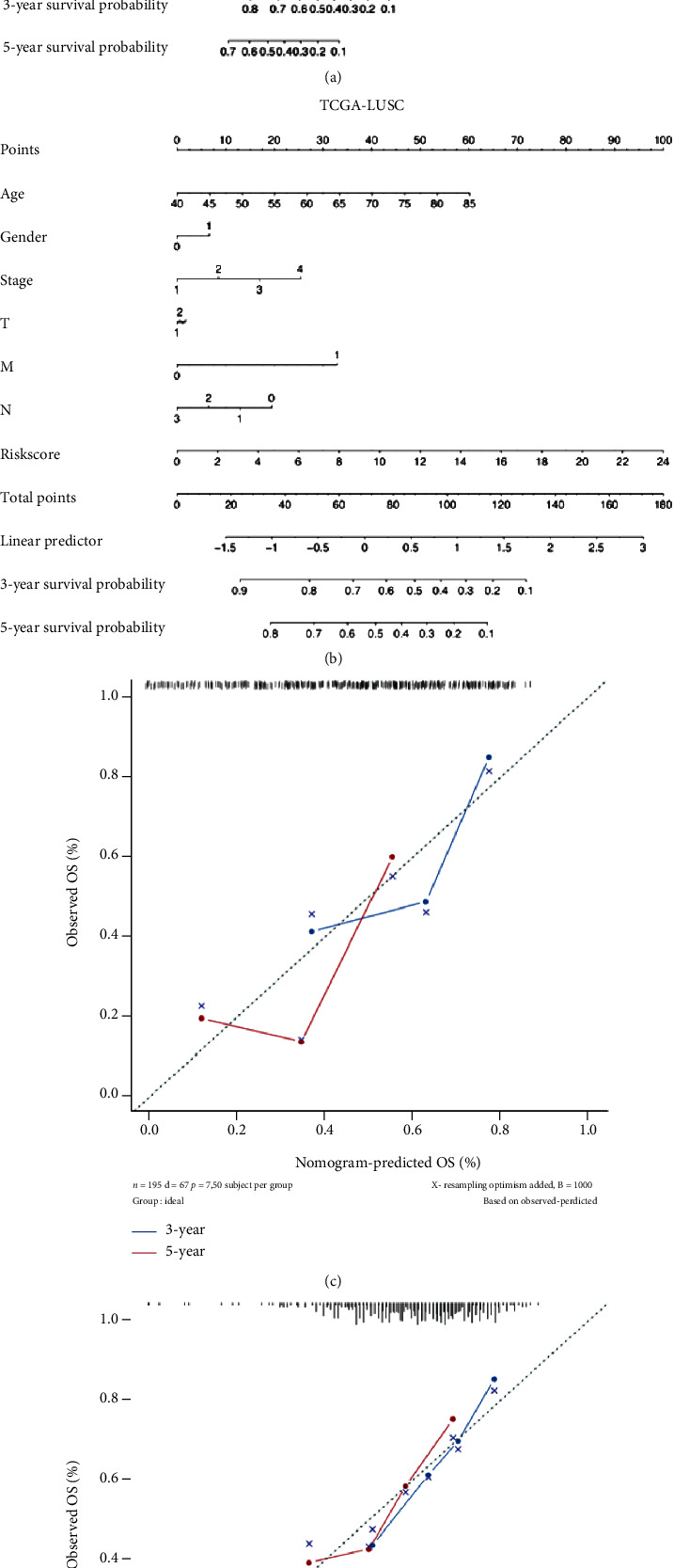
Development of LCSG-integrated nomograms and validation of predictive accuracy. The nomograms predicting 3- and 5-year overall survival for (a) LUAD and (b) LUSC patients. The calibration curve for predicting (c) LUAD and (d) LUSC patient survival at 3 years and 5 years.

To suggest a translational application of LCSG expression in lung cancer survival, we constructed LCSG-specific nomogram prediction models for LUAD and LUSC, incorporating age, sex, and tumour-node-metastasis (TNM) stage to quantitatively determine individual risk. As shown in the nomograms, the 3- and 5-year OS probabilities can be calculated based on the selected variables for LUAD and LUSC (Figures 6(a) and 6(b)). The actual and predicted values of 3- and 5-year OS were measured by calibration curves, showing acceptable consistency in both the LUAD and LUSC (Figures 6(c) and 6(d)) cohorts.

## 4. Discussion

Some genes have a biological role in the development or prevention of cancer, and their abnormal functions can increase the risk of cancer in affected individuals; these genes are known as CSGs. We named genes associated with the risk of lung cancer LCSGs. Genes associated with susceptibility to NSCLC have been identified in previous studies. According to a GWAS, the SNP rs2736100 localizes to *CLPTM1* L-*TERT* and is linked to the risk of lung cancer [15, 16]. Another case–control study showed that *ERCC3* could be regarded as an LCSG [17]. Hundreds of genes are considered to be associated with lung cancer susceptibility. However, how the expression of these genes affects lung cancer prognosis is unknown. Further mining the role of LCSGs in treatment could extend the role of CSGs in translational medicine, for example, multiple gene-based lung cancer prognosis.

In this study, we analysed the gene expression of the currently identified LCSGs in the TCGA-LUAD and TCGA-LUSC cohorts and their correlation with clinical data. Among the LCSGs, 21 genes and 13 genes were related to the survival of TCGA-LUAD and TCGA-LUSC, respectively. We further used LASSO regression to develop prognostic markers for the TCGA-LUAD and TCGA-LUSC cohorts, resulting in 10 genes and 7 genes, respectively. We divided patient survival outcomes into high-risk and low-risk groups based on the risk score established by integrating each patient's mRNA expression levels. This model was validated. Currently, gene signatures related to the clinical outcomes of NSCLC have been reported. Li et al. [18] developed a four-gene prognostic marker for LUSC, and LUAD has a sixteen-gene predictive marker, as reported by Ma et al. [19]. Beyond genes selected only by survival data, gene signatures have been developed integrating biological factors. For example, a glycolysis-related nine-gene signature [20] and immune-related fourteen-gene signature [21] for LUAD, an autophagy-related six-gene prognostic signature for both LUAD and LUSC [22, 23], and a seven-gene signature for lung cancer linked to smoking [24] have been reported. These genetic traits explain the importance of distinct biological processes in lung cancer prognosis, yet there are limited studies on LCSGs in lung cancer prognosis. Given the maturity of LCSG detection, we first constructed a lung cancer prognostic model based on LCSGs, which is expected to extend the translational value of LCSG testing at the time of secondary prevention.

The potential systematic impact of LCSGs on tumour metastasis and prognosis is unknown. We applied bioinformatics approaches to reveal the main biological signalling pathways affected by LCSGs. Interestingly, in the independent functional analysis of LUAD and LUSC, “acetylcholine-gated cation-selective channel activity,” “acetylcholine receptor activity,” and “excitatory extracellular ligand - gated ion channel activity” in the GO term molecular function category and “rheumatoid arthritis” in KEGG were shared in both groups. Tobacco usage is the most common cause of lung cancer, and nicotinic acetylcholine receptors are key components involved in cancer signalling [25]. This finding suggested that environmental cigarette smoking plus the vulnerability of the ion channel of an individual could be a powerful trigger for both LUAD and LUSC. Another KEGG term suggests that rheumatoid arthritis-related gene dysfunction may increase the risk of lung cancer, which is consistent with prior studies [26–28]. The conversion of epithelial cells to mesenchymal cells or mesenchymal-epithelial transition is a biological process that is often involved in carcinogenesis and metastasis. Negative regulation of epithelial cell differentiation was found to be one of the common pathways affected by differentially expressed genes in both LUAD and LUSC, suggesting that LCSGs could affect metastasis-associated pathways. These findings provide potential methods for LCSG-targeting drugs in cancer prevention and early metastasis intervention in populations harbouring this category of LCSGs.

A clinical nomogram is a graphical calculation tool for quantitatively assessing an individual's risk by assigning points to various factors from clinical information and summing all the points to a value representing the possibility of an outcome [29–31]. For further potential clinical application of the CSGs, we developed nomograms based on the LCSG risk scores and clinical information to predict individual prognostic outcomes. Our models show that in addition to traditional clinicopathological characteristics (e.g., age, sex, TNM stage, and tumour size), risk scores based on the LCSGs can be included as predictors of lung cancer prognosis. We show that nomograms containing the risk score generated by the expression of 10 and 7 LCSGs can predict the possibilities of 3- and 5-year survival in patients with LUAD and LUSC, respectively. This suggests that CSGs could be used to improve clinical prognostication.

There are some limitations to this study. Oncogenetic counselling usually involves monitoring peripheral blood for gene mutations and does not involve gene expression. Therefore, unless an additional test is performed, the model cannot be used based on routine information. Second, genetic alteration of LCSGs may not affect gene expression. Third, the link between germline mutation in normal tissue and gene expression in cancer needs further study. The contribution of the risk score to lung cancer risk is limited. Although this model needs to be validated in an independent dataset, it is the first analysis of how LCSG expression potentially mediates metastasis and affects prognosis. Establishment of a model or biological experiment validation of how genetic germline mutation links to gene expression could add more translational value of the presented studies.

## 5. Conclusions

In summary, using the data from TCGA-LUAD and TCGA-LUSC cohorts, we created a risk score based on LCSG expression. Our findings suggest that a set of LCSGs can be used as an independent predictor of the risk of metastasis and prognosis, a component of clinical nomograms, and targets for personalized cancer prevention.

## Figures and Tables

**Table 1:** The full name, genomic location, other associated diseases, and gene coefficients in the model.

Cancer type	Gene symbol	Full name	Genomic location^∗^	Other associated diseases	Risk coefficient
LUAD					
	*EPHX1*	Epoxide hydrolase 1	Chr 1	Hypercholanaemia, familial, and eclampsia	−0.16790078
	*PRDM2*	PR/SET domain 2	Chr 1	Retinoblastoma, Wilms tumour 5	−0.02219008
	*ABHD16A*	Abhydrolase domain containing 16A	Chr 6	Coronary artery aneurysm and lynch syndrome	−0.76723529
	*VEGFC*	Vascular endothelial growth factor C	Chr 4	Lymphatic malformation 4 and hereditary lymphedema id	0.28424844
	*EXO1*	Exonuclease 1	Chr 1	Werner syndrome and Aicardi-Goutieres syndrome	0.06614392
	*ABCA1*	ATP binding cassette subfamily A member 1	Chr 9	Tangier disease and Hypoalphalipoproteinemia	−0.07512348
	*DNAJB4*	DnaJ heat shock protein family (Hsp40) member B4	Chr 1	Oculopharyngeal muscular dystrophy	0.16479705
	*KRT8*	Keratin 8	Chr 12	Liver cirrhosis and cryptogenic cirrhosis	0.17566034
	*HLA-DOB*	Major histocompatibility complex, class II, DO Beta	Chr 6	Duodenal obstruction and systemic lupus erythematosus	−0.24952808
	*REXO4*	REX4 homologue, 3′-5′ exonuclease	Chr 9	Conjunctival pigmentation and uterine inversion	0.35525721
LUSC					
	*DCBLD1*	Discoidin, CUB and LCCL domain containing 1	Chr 6	—	0.3994102
	*HYKK*	Hydroxylysine kinase	Chr 15	Tobacco addiction	−0.1806394
	*SLC17A8*	Solute carrier family 17 member 8	Chr 12	Deafness, autosomal dominant 25 and autosomal dominant nonsyndromic sensorineural deafness type DFNA	1.38588891
	*HNF1B*	HNF1 Homeobox B	Chr 17	Renal cysts and diabetes syndrome and Hnf1b-related autosomal dominant tubulointerstitial kidney disease	0.13164075
	*ACE*	Angiotensin I converting enzyme	Chr 17	Microvascular complications of diabetes 3 and renal tubular dysgenesis	0.36932781
	*DAB2IP*	DAB2 interacting protein	Chr 9	Medulloblastoma and arteriosclerosis	0.05801815
	*FOXE1*	Forkhead box E1	Chr 9	Hypothyroidism, thyroidal, or athyroidal, with spiky hair and cleft palate and thyroid cancer	−0.0386071

^∗^Chr: chromosome. Information on genomic location and associated diseases were retrieved from the GeneCards (https://www.genecards.org).

## Data Availability

The reported data were obtained from the Genome-wide association studies (GWAS) catalogue (https://www.ebi.ac.uk/gwas/), The Cancer Genome Atlas (TCGA) Genomic Data Commons (GDC) (2019-12-06), and the Broad Institute Cancer Cell Line Encyclopedia (CCLE) database.

## Abbreviations

CCLE: Cancer Cell Line Encyclopedia
CI: Confidence interval
CSG: Cancer susceptibility gene
GDC: Genomic Data Commons
GSEA: Gene Set Enrichment Analysis
GWAS: Genome-wide association studies
HR: Hazard ratio
KEGG: Kyoto Encyclopedia of Genes and Genomes
LASSO: Least absolute shrinkage and selection operator
LCSG: Lung cancer susceptibility gene
LUAD: Lung adenocarcinoma
LUSC: Lung squamous cell carcinoma
NSCLC: Non-small cell lung cancer
OS: Overall survival
RPKM: Reads per kilobase of transcript, per million mapped reads
ROC: Receiver operating characteristic
SNP: Single-nucleotide polymorphism
STRING: Search Tool for the Retrieval of Interacting Genes/Proteins
TCGA: The Cancer Genome Atlas
TNM stage: Tumour-node-metastasis stage
XRCC: X-ray repair cross-complementation.

## Supplementary Materials

Supplementary Materials Figure S1: the impact of LCSGs on lung cancer survival. Meta-analysis of LCSG expression and the pooled HRs of OS in LUAD (a) and LUSC (b). A Venn diagram indicates common survival-associated LCSGs in both histologic types. Figure S2: establishment of the LCSG-specific signature and distribution of risk scores in each cohort. A machine learning approach, the least absolute shrinkage and selection operator (LASSO), was used to select the optimal number of genes for the risk score for TCGA-LUAD (a) and TCGA-LUSC (c). The LASSO coefficient of the genes in TCGA-LUAD (b) and TCGA-LUSC (d). The risk score and survival time distribution of each patient in TCGA-LUAD (e) and TCGA-LUSC (f) cohorts. Figure S3: validation of the LCSG-specific signature. Gene expression profiles of the LCSG-specific signature for TCGA-LUAD (a) and TCGA-LUSC (b) in the validation set. The risk score and survival time distributions of each patient in the TCGA-LUAD (c) and TCGA-LUSC (d) cohorts of the validation set. Table S1: potential lung cancer susceptibility genes identified in genome-wide association studies and a literature review. Table S2: lung cancer susceptibility genes associated with lung cancer survival in TCGA cohorts.
