# Supplementary material for: Potential Impact of Cancer Susceptibility Genes on Lung Cancer Metastasis
Source: J Oncol. 2022 Apr 18;2022:1516946. doi: 10.1155/2022/1516946 (PMC9038395; doi:10.1155/2022/1516946)
Supplement: Supplementary Materials — Figure S1: the impact of LCSGs on lung cancer survival. Meta-analysis of LCSG expression and the pooled HRs of OS in LUAD (a) and LUSC (b). A Venn diagram indicates common survival-associated LCSGs in both histologic types. Figure S2: establishment of the LCSG-specific signature and distribution of risk scores in each cohort. A machine learning approach, the least absolute shrinkage and selection operator (LASSO), was used to select the optimal number of genes for the risk score for TCGA-LUAD (a) and TCGA-LUSC (c). The LASSO coefficient of the genes in TCGA-LUAD (b) and TCGA-LUSC (d). The risk score and survival time distribution of each patient in TCGA-LUAD (e) and TCGA-LUSC (f) cohorts. Figure S3: validation of the LCSG-specific signature. Gene expression profiles of the LCSG-specific signature for TCGA-LUAD (a) and TCGA-LUSC (b) in the validation set. The risk score and survival time distributions of each patient in the TCGA-LUAD (c) and TCGA-LUSC (d) cohorts of the validation set. Table S1: potential lung cancer susceptibility genes identified in genome-wide association studies and a literature review. Table S2: lung cancer susceptibility genes associated with lung cancer survival in TCGA cohorts. [file 1516946.f1.zip › Figure S2.pdf]

Figure S2

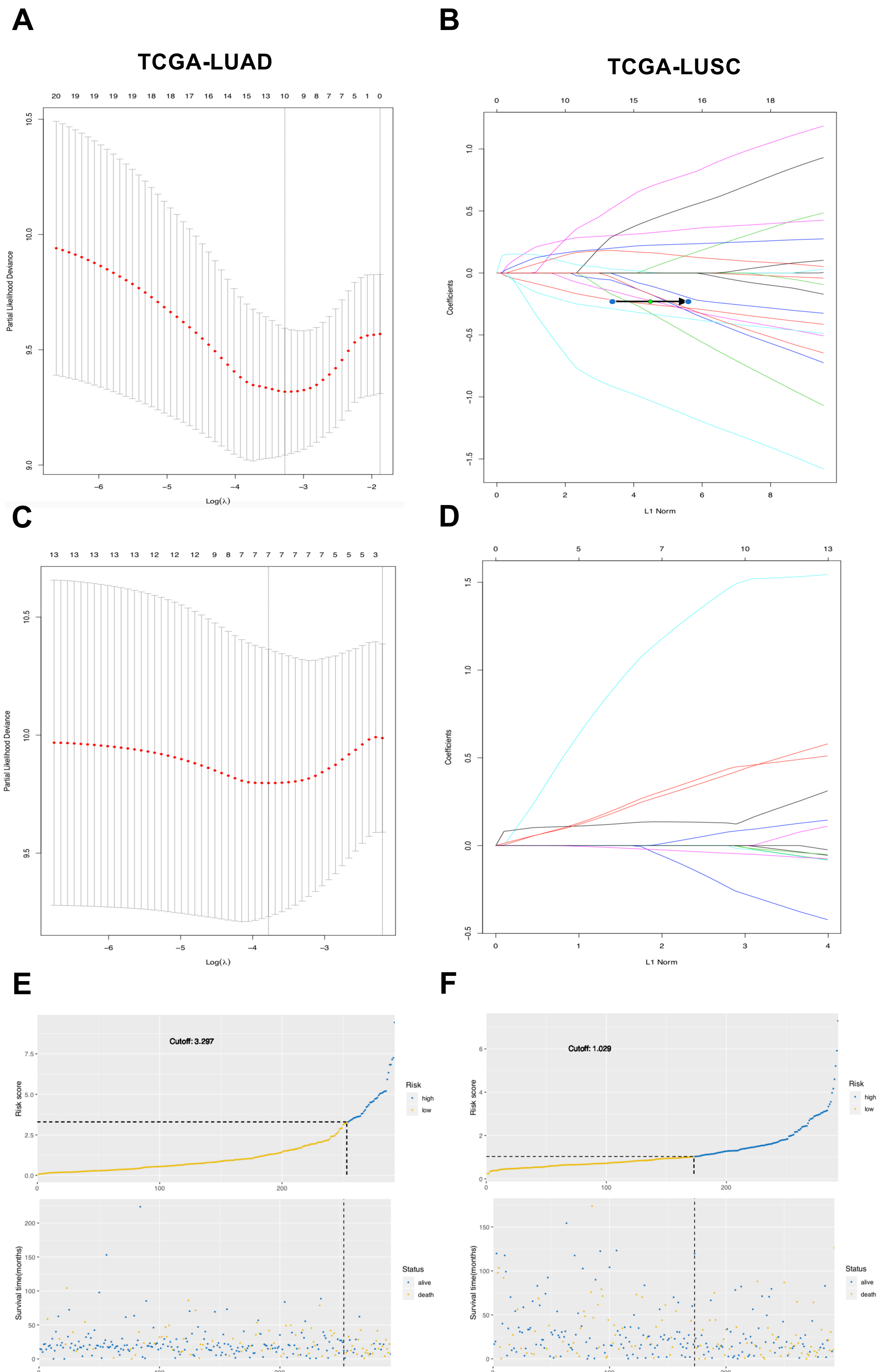

Figure S2. Establishment of the LCSG-specific signature and distribution of risk scores in each cohort. A machine learning approach, the least absolute shrinkage and selection operator (LASSO), was used to select the optimal number of genes for the risk score for TCGA-LUAD (a) and TCGA-LUSC (c). The LASSO coefficient of the genes in TCGA-LUAD (b) and TCGA-LUSC (d). The risk score and survival time distribution of each patient in the TCGA-LUAD (e) and TCGA-LUSC (f) cohorts.
