# Supplementary material for: Potential Impact of Cancer Susceptibility Genes on Lung Cancer Metastasis
Source: J Oncol. 2022 Apr 18;2022:1516946. doi: 10.1155/2022/1516946 (PMC9038395; doi:10.1155/2022/1516946)
Supplement: Supplementary Materials — Figure S1: the impact of LCSGs on lung cancer survival. Meta-analysis of LCSG expression and the pooled HRs of OS in LUAD (a) and LUSC (b). A Venn diagram indicates common survival-associated LCSGs in both histologic types. Figure S2: establishment of the LCSG-specific signature and distribution of risk scores in each cohort. A machine learning approach, the least absolute shrinkage and selection operator (LASSO), was used to select the optimal number of genes for the risk score for TCGA-LUAD (a) and TCGA-LUSC (c). The LASSO coefficient of the genes in TCGA-LUAD (b) and TCGA-LUSC (d). The risk score and survival time distribution of each patient in TCGA-LUAD (e) and TCGA-LUSC (f) cohorts. Figure S3: validation of the LCSG-specific signature. Gene expression profiles of the LCSG-specific signature for TCGA-LUAD (a) and TCGA-LUSC (b) in the validation set. The risk score and survival time distributions of each patient in the TCGA-LUAD (c) and TCGA-LUSC (d) cohorts of the validation set. Table S1: potential lung cancer susceptibility genes identified in genome-wide association studies and a literature review. Table S2: lung cancer susceptibility genes associated with lung cancer survival in TCGA cohorts. [file 1516946.f1.zip › Table S1 Potential lung cancer susceptibility genes identified in genome-wide association studies and literature review..pdf]

Table 1

Gene_name	Cancer_type	PMID	Resource
TERT	lung cancer	28789383;29928145; 24390616	Literature Review
CLPTM1L	lung cancer	29110844	Literature Review
ALDH2	lung cancer	20093384	Literature Review
FAS	lung cance	27103831 ; 17708363	Literature Review
GSTM1	lung cancer	29604112 ; 24682953 ; 23803127	Literature Review
SULT1A1	lung cancer	29110586 ; 19350537	Literature Review
NQO1	lung cancer	12163326	Literature Review
ACE	lung cancer	27328622 ; 25605160	Literature Review
CAV1	lung cancer	29190968	Literature Review
IL13	lung cancer	30671474	Literature Review
XRCC1	lung cancer	27819744;27323144 ;29110586;25433331; 25146668	Literature Review
CYP1B1	lung cancer	26283052;24989113 ;29110844 ;29938532	Literature Review
HIF1A	lung cancer	28302216	Literature Review
MMP3	lung cancer	22587600 ; 15528217	Literature Review
LAPTM4B	lung cancer	15968325	Literature Review
OGG1	lung cancer	9587483 ; 29209987	Literature Review
APEX1	lung cancer	28827732 ;25156607 ; 23065211	Literature Review
CASP3	lung cancer	27723786	Literature Review
ERCC4	lung cancer	18068852	Literature Review
MTHFR	lung cancer	25422209	Literature Review
RTEL1	lung cancer	27765928 ; 29924316	Literature Review
CCL2	lung cancer	27145753	Literature Review
ERCC1	lung cancer	24841208	Literature Review
ERCC3	lung cancer..	16835333	Literature Review
GSTM3	lung cancer	25040976	Literature Review
IL17A	lung cancer	26073462;25469655;	Literature Review
MGMT	lung cancer	24390665 ; 17285603	Literature Review
NAT2	lung cancer	25422202;25005845	Literature Review
CD44	lung cancer	30860617	Literature Review
DDB2	lung cancer	16522664	Literature Review
EGFR	lung cancer	24495289	Literature Review
ERCC2	lung cancer	27819744;16835333	Literature Review
EXO1	lung cancer	19331228	Literature Review
HAVCR2	lung cancer	23359271	Literature Review

HDAC9	lung adenocarcinoma	24650256	Literature Review
ICOS	lung cancer	21669243	Literature Review
MBD4	lung cancer	18495292 ; 16803845	Literature Review
MMP9	lung cancer	23467666	Literature Review
MUTYH	lung cancer	29209987	Literature Review
STAT3	lung cancer	21948749;24382554 ; 30689078	Literature Review
TOX3	lung cancer	27486757	Literature Review
TP63	lung cancer	31204706;27063419 ;24466311;24377560;24092572;22367405;20871597	Literature Review
WWOX	lung cancer	22693020	Literature Review
XPC	lung cancer	28540485 ; 23381646;24375193	Literature Review
ABCA1	lung cancer	25104170	Literature Review
ABCC1	lung cancer	21736601	Literature Review
AGER	lung cancer	26782423	Literature Review
ALK	lung cancer	23273562	Literature Review
ATP2A2	lung cancer	17116488	Literature Review
BCL2	lung cancer	23977251 ; 26311051	Literature Review
BIRC5	lung cancer	23232114	Literature Review
BTNL2	lung cancer	28025329	Literature Review
CASP5	lung cancer	19203830	Literature Review
CHRNA6	lung cancer	21831805	Literature Review
CYP1A2	lung cancer	25472037	Literature Review
DDB2	lung cancer	16522664	Literature Review
EPHX1	lung cancer	26770388	Literature Review
GSTT1	lung cancer	23803127	Literature Review
HSPA1B	lung cancer	22037874	Literature Review
IL4	lung cancer	25450271	Literature Review
LPP	lung cancer	30621612	Literature Review
MAD1L1	lung cancer	20516147	Literature Review
MAD2L1	lung cancer	20516147	Literature Review
NAT1	lung adenocarcinoma	11266080	Literature Review
NME1	lung cancer	17688968	Literature Review
NRG1	lung cancer	28604730	Literature Review
POT1	lung cancer	19285750	Literature Review
POU5F1	lung cancer	26824036	Literature Review
PTCH1	lung cancer	30032850	Literature Review
SOD2	lung cancer	23990443	Literature Review

VEGFC	lung cancer	24146275	Literature Review
XPA	lung cancer	27460688;29516319	Literature Review
ABHD16A	lung cancer	18978787	Literature Review
ACACA	lung squamous cell c	28546520	Literature Review
AXIN2	lung cancer	28378643;28827732	Literature Review
C18orf54	lung cancer	19622765	Literature Review
C6orf10	lung cancer	28604730	Literature Review
CDK5	lung cancer	19343042	Literature Review
CHRNA3	lung cancer	26831765 ; 29609626 ; 28827732 ; 25806352 ; 29110844 ; 23023782	Literature Review
CHRNA5	lung cancer	29609626 ; 28827732 ; 23314339 ; 27050379 ; 26693074	Literature Review
CHRNB3	lung cancer	21831805	Literature Review
CHRNB4	lung cancer	21747048;26942719	Literature Review
CYP2A6	lung cancer	21747048	Literature Review
DAB2IP	lung cancer	22046421	Literature Review
DNAJA4	lung cancer	28604730	Literature Review
ERCC6	lung cancer	18789574	Literature Review
GAB1	lung cancer	29212272	Literature Review
GPC5	lung cancer	22236185	Literature Review
GTF2H1	lung cancer	18692935	Literature Review
IL18	lung cancer	28002583	Literature Review
IREB2	lung cancer	28181565 ; 26310313	Literature Review
JUN	lung cancer	22189957	Literature Review
KLF6	lung cancer	17223258	Literature Review
LRP5	lung cancer	27698794	Literature Review
MDC1	lung cancer	25198518	Literature Review
MMP1	lung cancer	22965879	Literature Review
MPO	lung cancer	24786234	Literature Review
PARK2	lung cancer	25640678	Literature Review
POLI	lung cancer	15609317	Literature Review
PRDM2	lung cancer	17693662	Literature Review
RASSF1	lung cancer	19821102 ; 16125301	Literature Review
REV3L	lung cancer	28827732	Literature Review
RGS17	lung cancer	19351763	Literature Review
SMARCA2	lung cancer	21478907	Literature Review
SUV39H2	lung cancer	16774942	Literature Review
VDR	lung cancer	28411367	Literature Review

XRN2	lung cancer	19915612	Literature Review
EGFR	Non-small cell lung c	16258541	Previous Report
BAP1	lung cancer (adenoca	21874003	Previous Report
TERT	Lung adenocarcinom	20700438	GWAS Catalog
TP63	Lung adenocarcinom	20871597	GWAS Catalog
TERT	Lung adenocarcinom	20871597	GWAS Catalog
XXYLT1	Non-small cell lung c	20876614	GWAS Catalog
EIF4E2	Non-small cell lung c	21079520	GWAS Catalog
DYNC2H1 - LOC	Small-cell lung cance	21118971	GWAS Catalog
HYKK	Lung cancer	18385676	GWAS Catalog
HYKK	Lung cancer	18385738	GWAS Catalog
HYKK	Lung cancer	18780872	GWAS Catalog
APOM, BAG6	Lung cancer	18978787	GWAS Catalog
CLPTM1L	Lung cancer	18978787	GWAS Catalog
CHRNA3	Lung cancer	18978787	GWAS Catalog
CHRNA3	Lung cancer	18978790	GWAS Catalog
HYKK	Lung cancer	19654303	GWAS Catalog
MIR4457 - CLPTI	Lung cancer	19654303	GWAS Catalog
APOM, BAG6	Lung cancer	19654303	GWAS Catalog
TP63	Lung cancer	21725308	GWAS Catalog
TERT	Lung cancer	21725308	GWAS Catalog
LOC105370113	Lung cancer	21725308	GWAS Catalog
MTMR3	Lung cancer	21725308	GWAS Catalog
TERT	Lung adenocarcinom	22797724	GWAS Catalog
TP63	Lung adenocarcinom	22797724	GWAS Catalog
BPTF	Lung adenocarcinom	22797724	GWAS Catalog
LOC101929163, E	Lung adenocarcinom	22797724	GWAS Catalog
RAD52	Lung cancer	22899653	GWAS Catalog
TERT	Lung adenocarcinom	19836008	GWAS Catalog
CHRNA3	Lung adenocarcinom	19836008	GWAS Catalog
CLPTM1L	Lung adenocarcinom	19836008	GWAS Catalog
APOM, BAG6	Lung adenocarcinom	19836008	GWAS Catalog
TRF-GAA3-1 - TI	Lung adenocarcinom	19836008	GWAS Catalog
LRFN2	Multiple cancers (lun	23103227	GWAS Catalog
DNAH11	Multiple cancers (lun	23103227	GWAS Catalog
DNAH11	Multiple cancers (lun	23103227	GWAS Catalog

TP63	Lung cancer	23143601	GWAS Catalog
TERT	Lung cancer	23143601	GWAS Catalog
VTI1A	Lung cancer	23143601	GWAS Catalog
RNU6-253P - DC	Lung cancer	23143601	GWAS Catalog
HLA-DRB9 - HL	Lung cancer	23143601	GWAS Catalog
SLC17A8 - NR1H	Squamous cell carcin	23341777	GWAS Catalog
BRCA2	Cancer	27197191	GWAS Catalog
HNF1B	Cancer	27197191	GWAS Catalog
RPS2P1 - ASIP	Cancer	27197191	GWAS Catalog
RALY	Cancer	27197191	GWAS Catalog
DVL1 - MXRA8	Cancer (pleiotropy)	27197191	GWAS Catalog
NEK10	Cancer (pleiotropy)	27197191	GWAS Catalog
LOC100288146 -	Cancer (pleiotropy)	27197191	GWAS Catalog
TERT	Cancer (pleiotropy)	27197191	GWAS Catalog
LOC101928448 -	Cancer (pleiotropy)	27197191	GWAS Catalog
HLA-DQB1 - MT	Cancer (pleiotropy)	27197191	GWAS Catalog
FGFR2	Cancer (pleiotropy)	27197191	GWAS Catalog
LOC105369366 -	Cancer (pleiotropy)	27197191	GWAS Catalog
LOC105369463 -	Cancer (pleiotropy)	27197191	GWAS Catalog
KRT8	Cancer (pleiotropy)	27197191	GWAS Catalog
BRCA2	Cancer (pleiotropy)	27197191	GWAS Catalog
RAD51B	Cancer (pleiotropy)	27197191	GWAS Catalog
HNF1B	Cancer (pleiotropy)	27197191	GWAS Catalog
BABAM1 - ANK	Cancer (pleiotropy)	27197191	GWAS Catalog
FRY	Cancer (pleiotropy)	27197191	GWAS Catalog
TERT	Lung adenocarcinom	27393504	GWAS Catalog
FOXP4-AS1	Adenocarcinoma	26732429	GWAS Catalog
CDKN2B-AS1 - I	Adenocarcinoma	26732429	GWAS Catalog
ACVR1B	Adenocarcinoma	26732429	GWAS Catalog
TERT	EGFR mutation-posi	27501781	GWAS Catalog
HLA-DPB1 - HL	EGFR mutation-posi	27501781	GWAS Catalog
LOC101929163, I	EGFR mutation-posi	27501781	GWAS Catalog
TP63	EGFR mutation-posi	27501781	GWAS Catalog
BPTF	EGFR mutation-posi	27501781	GWAS Catalog
LINC01276 - FO	EGFR mutation-posi	27501781	GWAS Catalog
FOXP4-AS1	Lung cancer	26732429	GWAS Catalog

CDKN2B-AS1 - I	Lung cancer	26732429	GWAS Catalog
ACVR1B	Lung cancer	26732429	GWAS Catalog
BRCA2	Lung cancer	24880342	GWAS Catalog
CHEK2	Lung cancer	24880342	GWAS Catalog
TP63	Lung cancer	24880342	GWAS Catalog
SECISBP2L - CO	Lung adenocarcinom	28604730	GWAS Catalog
GALK2	Lung adenocarcinom	28604730	GWAS Catalog
GALK2	Lung adenocarcinom	28604730	GWAS Catalog
FGF7, FAM227B	Lung adenocarcinom	28604730	GWAS Catalog
TEKT5	Lung adenocarcinom	28604730	GWAS Catalog
CYP2A6	Lung adenocarcinom	28604730	GWAS Catalog
TP63	Lung adenocarcinom	28604730	GWAS Catalog
MORF4L1	Lung adenocarcinom	28604730	GWAS Catalog
DTWD1 - RLIMP	Lung adenocarcinom	28604730	GWAS Catalog
RNU6-253P - DC	Lung adenocarcinom	28604730	GWAS Catalog
LOC107984734 -	Lung adenocarcinom	28604730	GWAS Catalog
LOC105369803	Lung adenocarcinom	28604730	GWAS Catalog
MICG - HCG4P8	Lung adenocarcinom	28604730	GWAS Catalog
MPZL2	Lung adenocarcinom	28604730	GWAS Catalog
NRG1	Lung adenocarcinom	28604730	GWAS Catalog
ADAMTS7	Lung adenocarcinom	28604730	GWAS Catalog
OBFC1 - LOC102	Lung adenocarcinom	28604730	GWAS Catalog
DNAJB4	Lung adenocarcinom	28604730	GWAS Catalog
MTAP	Lung adenocarcinom	28604730	GWAS Catalog
CDKN2B-AS1	Lung adenocarcinom	28604730	GWAS Catalog
AK5	Lung adenocarcinom	28604730	GWAS Catalog
RPL18P11 - LOC	Lung adenocarcinom	28604730	GWAS Catalog
TERT	Lung adenocarcinom	28604730	GWAS Catalog
CHRNA5	Lung adenocarcinom	28604730	GWAS Catalog
DCTN4	Lung adenocarcinom	28604730	GWAS Catalog
CHRNA5	Lung cancer	27393504	GWAS Catalog
TERT	Lung cancer	27393504	GWAS Catalog
AK5	Lung cancer	28604730	GWAS Catalog
DNAJB4	Lung cancer	28604730	GWAS Catalog
LOC107984997 -	Lung cancer	28604730	GWAS Catalog
ZAK	Lung cancer	28604730	GWAS Catalog

SIX3 - LOC10192	Lung cancer	28604730	GWAS Catalog
LOC102800447	Lung cancer	28604730	GWAS Catalog
LOC105377262	Lung cancer	28604730	GWAS Catalog
CLPTM1L	Lung cancer	28604730	GWAS Catalog
RNASET2 - LOC	Lung cancer	28604730	GWAS Catalog
SLC17A1, SLC17	Lung cancer	28604730	GWAS Catalog
LOC107986581 -	Lung cancer	28604730	GWAS Catalog
HIST1H4H - TRX	Lung cancer	28604730	GWAS Catalog
BTN3A1, BTN3A	Lung cancer	28604730	GWAS Catalog
VN1R14P	Lung cancer	28604730	GWAS Catalog
TRM-CAT4-3 - T	Lung cancer	28604730	GWAS Catalog
LINC00240	Lung cancer	28604730	GWAS Catalog
TRT-AGT6-1 - TI	Lung cancer	28604730	GWAS Catalog
LOC105375001	Lung cancer	28604730	GWAS Catalog
TRT-AGT2-2 - TI	Lung cancer	28604730	GWAS Catalog
RSL24D1P1 - TR	Lung cancer	28604730	GWAS Catalog
RNU7-26P - TRX	Lung cancer	28604730	GWAS Catalog
ZNF165 - ZNF60	Lung cancer	28604730	GWAS Catalog
PGBD1 - ZSCAN	Lung cancer	28604730	GWAS Catalog
ZSCAN23	Lung cancer	28604730	GWAS Catalog
TRF-GAA4-1 - TI	Lung cancer	28604730	GWAS Catalog
TRM-CAT3-2 - K	Lung cancer	28604730	GWAS Catalog
OR2J2 - OR2J4P	Lung cancer	28604730	GWAS Catalog
LOC105375005, I	Lung cancer	28604730	GWAS Catalog
SUMO2P1 - MOC	Lung cancer	28604730	GWAS Catalog
3.8-1.5 - HCG4	Lung cancer	28604730	GWAS Catalog
ZNRD1	Lung cancer	28604730	GWAS Catalog
TRIM26	Lung cancer	28604730	GWAS Catalog
UBQLN1P1 - MIC	Lung cancer	28604730	GWAS Catalog
HLA-E - GNL1	Lung cancer	28604730	GWAS Catalog
PPP1R18	Lung cancer	28604730	GWAS Catalog
DDR1	Lung cancer	28604730	GWAS Catalog
MUC22	Lung cancer	28604730	GWAS Catalog
C6orf15 - PSORS	Lung cancer	28604730	GWAS Catalog
HCG27 - HLA-C	Lung cancer	28604730	GWAS Catalog
HCP5 - HCG26	Lung cancer	28604730	GWAS Catalog

APOM, BAG6	Lung cancer	28604730	GWAS Catalog
MSH5, MSH5-SA	Lung cancer	28604730	GWAS Catalog
SLC44A4	Lung cancer	28604730	GWAS Catalog
TNXB	Lung cancer	28604730	GWAS Catalog
LOC101929163	Lung cancer	28604730	GWAS Catalog
BTNL2 - HLA-DI	Lung cancer	28604730	GWAS Catalog
HLA-DQA1, LOC	Lung cancer	28604730	GWAS Catalog
HLA-DQB2 - HL	Lung cancer	28604730	GWAS Catalog
CHRNA2 - EPHX	Lung cancer	28604730	GWAS Catalog
CDKN2B-AS1	Lung cancer	28604730	GWAS Catalog
FAM178A	Lung cancer	28604730	GWAS Catalog
MPZL2	Lung cancer	28604730	GWAS Catalog
WNK1	Lung cancer	28604730	GWAS Catalog
BRCA2	Lung cancer	28604730	GWAS Catalog
PDS5B	Lung cancer	28604730	GWAS Catalog
SEMA6D	Lung cancer	28604730	GWAS Catalog
SECISBP2L - CO	Lung cancer	28604730	GWAS Catalog
CHRNA5	Lung cancer	28604730	GWAS Catalog
RPL18P11 - LOC	Lung cancer	28604730	GWAS Catalog
ADAMTS7	Lung cancer	28604730	GWAS Catalog
MORF4L1	Lung cancer	28604730	GWAS Catalog
CYP2A6	Lung cancer	28604730	GWAS Catalog
CLPTM1L	Squamous cell lung c	28604730	GWAS Catalog
LRRC16A	Squamous cell lung c	28604730	GWAS Catalog
SCGN	Squamous cell lung c	28604730	GWAS Catalog
HIST1H2AC - HI	Squamous cell lung c	28604730	GWAS Catalog
LOC101928743	Squamous cell lung c	28604730	GWAS Catalog
BTN3A3, BTN3A	Squamous cell lung c	28604730	GWAS Catalog
ZNF322	Squamous cell lung c	28604730	GWAS Catalog
TRT-AGT6-1 - TI	Squamous cell lung c	28604730	GWAS Catalog
TRK-TTT6-1 - Z	Squamous cell lung c	28604730	GWAS Catalog
LOC105375001	Squamous cell lung c	28604730	GWAS Catalog
TRD-GTC3-1 - T	Squamous cell lung c	28604730	GWAS Catalog
OR2B2 - OR2W6	Squamous cell lung c	28604730	GWAS Catalog
OR2W2P - OR2B	Squamous cell lung c	28604730	GWAS Catalog
PGBD1	Squamous cell lung c	28604730	GWAS Catalog

ZSCAN12	Squamous cell lung c	28604730	GWAS Catalog
GPX6	Squamous cell lung c	28604730	GWAS Catalog
TRF-GAA4-1 - TI	Squamous cell lung c	28604730	GWAS Catalog
TRM-CAT3-2 - K	Squamous cell lung c	28604730	GWAS Catalog
OR2N1P - OR2J2	Squamous cell lung c	28604730	GWAS Catalog
LOC105375005, I	Squamous cell lung c	28604730	GWAS Catalog
3.8-1.5 - HCG4	Squamous cell lung c	28604730	GWAS Catalog
HLA-K - HLA-U	Squamous cell lung c	28604730	GWAS Catalog
ZNRD1	Squamous cell lung c	28604730	GWAS Catalog
TRIM26	Squamous cell lung c	28604730	GWAS Catalog
UBQLN1P1 - MI(	Squamous cell lung c	28604730	GWAS Catalog
HLA-E - GNL1	Squamous cell lung c	28604730	GWAS Catalog
VARS2	Squamous cell lung c	28604730	GWAS Catalog
MUC22	Squamous cell lung c	28604730	GWAS Catalog
HCG27 - HLA-C	Squamous cell lung c	28604730	GWAS Catalog
HCP5 - HCG26	Squamous cell lung c	28604730	GWAS Catalog
APOM, BAG6	Squamous cell lung c	28604730	GWAS Catalog
C6orf48 - NEU1	Squamous cell lung c	28604730	GWAS Catalog
TNXB	Squamous cell lung c	28604730	GWAS Catalog
NOTCH4 - LOC1(	Squamous cell lung c	28604730	GWAS Catalog
LOC101929163	Squamous cell lung c	28604730	GWAS Catalog
HLA-DQA1, LOC	Squamous cell lung c	28604730	GWAS Catalog
HLA-DQB2 - HL/	Squamous cell lung c	28604730	GWAS Catalog
RNU4-66P - RIM!	Squamous cell lung c	28604730	GWAS Catalog
REXO4	Squamous cell lung c	28604730	GWAS Catalog
WNK1	Squamous cell lung c	28604730	GWAS Catalog
FRY	Squamous cell lung c	28604730	GWAS Catalog
BRCA2	Squamous cell lung c	28604730	GWAS Catalog
PDS5B	Squamous cell lung c	28604730	GWAS Catalog
CHRNA3	Squamous cell lung c	28604730	GWAS Catalog
LOC105370913	Squamous cell lung c	28604730	GWAS Catalog
CYP2A6	Squamous cell lung c	28604730	GWAS Catalog
LOC105375015 -	Small cell lung carci	28604730	GWAS Catalog
BRCA2	Small cell lung carci	28604730	GWAS Catalog
CHRNA5	Small cell lung carci	28604730	GWAS Catalog
LOC105370913	Small cell lung carci	28604730	GWAS Catalog

CHRNA3	Familial lung cancer	29924316	GWAS Catalog
LOC105373934	Familial lung adenoc	29924316	GWAS Catalog
LMCD1-AS1	Familial lung adenoc	29924316	GWAS Catalog
LCORL	Familial squamous c	29924316	GWAS Catalog
MIR31HG - KHSI	Familial squamous c	29924316	GWAS Catalog
LCORL	Familial squamous c	29924316	GWAS Catalog
CENPP	Familial squamous c	29924316	GWAS Catalog
KRT18P13 - FOX	Familial squamous c	29924316	GWAS Catalog
MKK7	lung cancer	27028764	Literature Review
