# Supplementary material for: Potential Impact of Cancer Susceptibility Genes on Lung Cancer Metastasis
Source: J Oncol. 2022 Apr 18;2022:1516946. doi: 10.1155/2022/1516946 (PMC9038395; doi:10.1155/2022/1516946)
Supplement: Supplementary Materials — Figure S1: the impact of LCSGs on lung cancer survival. Meta-analysis of LCSG expression and the pooled HRs of OS in LUAD (a) and LUSC (b). A Venn diagram indicates common survival-associated LCSGs in both histologic types. Figure S2: establishment of the LCSG-specific signature and distribution of risk scores in each cohort. A machine learning approach, the least absolute shrinkage and selection operator (LASSO), was used to select the optimal number of genes for the risk score for TCGA-LUAD (a) and TCGA-LUSC (c). The LASSO coefficient of the genes in TCGA-LUAD (b) and TCGA-LUSC (d). The risk score and survival time distribution of each patient in TCGA-LUAD (e) and TCGA-LUSC (f) cohorts. Figure S3: validation of the LCSG-specific signature. Gene expression profiles of the LCSG-specific signature for TCGA-LUAD (a) and TCGA-LUSC (b) in the validation set. The risk score and survival time distributions of each patient in the TCGA-LUAD (c) and TCGA-LUSC (d) cohorts of the validation set. Table S1: potential lung cancer susceptibility genes identified in genome-wide association studies and a literature review. Table S2: lung cancer susceptibility genes associated with lung cancer survival in TCGA cohorts. [file 1516946.f1.zip › Table S2 Lung cancer susceptibility genes associated with lung cancer survival in TCGA cohorts. (1).pdf]

Table S2 LCSGs significantly associated with the OS of TCGA-LUAD and TCGA-LUSC

LUAD			LUSC		
**Gene**	**Hazard Ratio**	**P Value**	**Gene**	**Hazard Ratio**	**P Value**
XXYLT1	1.48	0.0244	HYKK	0.56	0.04026
WWOX	0.59	0.03614	ANKLE1	0.61	0.03991
EPHX1	0.78	0.008	CHEK2	0.74	0.043
XRCC1	1.54	0.03979	FOXE1	0.9	0.04144
PRDM2	0.53	0.01952	AGER	1.13	0.03335
ABHD16A	0.48	0.02163	JUN	1.21	0.04147
VEGFC	1.33	0.00226	DAB2IP	1.3	0.02569
AK5	2.45	0.00233	AXIN2	1.3	0.02464
AGER	0.89	0.04313	ACE	1.34	0.04061
ERCC1	1.83	0.00111	KLF6	1.41	0.00352
NME1	1.33	0.03473	DCBLD1	1.41	0.01992
EXO1	1.4	0.00355	HNF1B	1.44	0.0452
ABCA1	0.73	0.03942	SLC17A8	3.06	0.00973
BIRC5	1.25	0.01083			
DNAJB4	1.48	0.00523			
C18orf54	1.79	0.02154			
KRT8	1.44	0.0002			
HLA.DOB	0.77	0.04911			
REXO4	1.8	0.02871			
MTHFR	0.6	0.00963			
DAB2IP	0.7	0.04612			
